# The Role of ZNF598 in Translational Quality Control: Mechanisms and Emerging Biological Functions

**DOI:** 10.3390/biology15161435

**Published:** 2026-08-20

**Authors:** Siyuan Wu, Zhiqian Liu, Guodong Chen

**Affiliations:** 1 Department of Obstetrics and Gynecology, School of Medicine, Hunan Normal University, Changsha 410013, China; 13637551920@163.com; 2 Center for Medical Genetics, Hunan Key Laboratory of Medical Genetics, School of Life Sciences, Central South University, Changsha 410078, China; liuzhiqian@csu.edu.cn; 3 Hunan Provincial Key Laboratory of Regional Hereditary Birth Defects Prevention and Control, Changsha Hospital for Maternal & Child Health Care Affiliated to Hunan Normal University, Changsha 410007, China

**Keywords:** ZNF598, ribosome-associated quality control, ribosome collision, GIGYF2, ubiquitination, translational stalling

## Abstract

During protein synthesis, ribosome stalling, collision, and aberrant elongation can lead to the accumulation of defective nascent polypeptides and compromise cellular homeostasis. To counteract such translational disturbances, eukaryotic cells have evolved a highly conserved translational quality control network, in which the ribosome-associated quality control (RQC) pathway plays a central role in the recognition and elimination of aberrant translation complexes. Zinc Finger Protein 598 (ZNF598), a key E3 ubiquitin ligase in mammalian cells, functions as an essential factor in the early recognition and signal transduction steps of the RQC pathway. Accumulating evidence indicates that ZNF598 senses aberrant translational states, and particularly in the context of ribosome collision, mediates site-specific ubiquitination of 40S ribosomal proteins, thereby promoting ribosome splitting, nascent chain clearance, and subsequent processing of defective mRNAs. Beyond its canonical role in RQC, ZNF598 has also been implicated in the translational repression of defective mRNAs, regulation of inflammatory signaling, antiviral responses, and control of toxic translation products associated with neurodegenerative disorders. In this review, we summarize the structural features, molecular mechanisms, regulatory networks, and physiological as well as pathological functions of ZNF598. We also discuss current controversies and future directions in the field, with the aim of providing a broader framework for understanding translational quality control and its therapeutic potential.

## Simple Summary

Protein production is essential for all living cells, but errors can occur during this process, leading to the formation of defective proteins that may damage cellular function. Cells therefore rely on surveillance systems to detect and remove these errors. This review focuses on a protein called Zinc Finger Protein 598, which serves as an important early sensor of problems that arise during protein production. We summarize current evidence showing that this protein recognizes abnormal protein-making machines, marks them for repair, and coordinates the removal of defective products and damaged genetic messages. Recent studies also indicate that it participates in controlling inflammation, defending against viral infection, and limiting the production of harmful proteins linked to neurological disorders. Together, these findings suggest that Zinc Finger Protein 598 plays a central role in maintaining cellular health and responding to stress. A better understanding of its functions may provide new opportunities for developing therapies for neurodegenerative diseases, infectious diseases, and other conditions associated with defects in protein quality control.

## 1. Introduction

Protein synthesis is a fundamental biological process that is essential for cellular function and organismal viability. The fidelity of translation is tightly linked to proteostasis and cellular integrity. However, under both physiological and pathological conditions, a variety of factors—including defective mRNAs, aberrant termination, rare codon enrichment, positively charged amino acid stretches, drug exposure, and diverse forms of cellular stress—can impair translational elongation and induce ribosome pausing, stalling, or the collision of multiple ribosomes on the same transcript [1,2,3]. If these aberrant translational events are not promptly recognized and resolved, the resulting accumulation of defective nascent chains and damaged mRNAs may trigger proteotoxic stress and disrupt cellular homeostasis.

To cope with such challenges, cells have evolved multiple layers of translational surveillance machinery, including no-go decay (NGD), non-stop decay (NSD), and ribosome-associated quality control (RQC) [1,2]. Among these pathways, RQC is primarily responsible for recognizing stalled or aberrant translation complexes and preventing the propagation of translational errors through ribosome splitting, nascent chain degradation, and defective mRNA turnover. In recent years, ribosome collision has emerged not only as a hallmark of translational dysfunction, but also as a key signal for the activation of downstream quality control pathways [3,4].

ZNF598 is a central E3 ubiquitin ligase in mammalian cells, and its yeast homolog is Hel2. Since its identification as a sensor of collided ribosomes, ZNF598 has become a major focus in the study of translational quality control. Available evidence indicates that ZNF598 converts aberrant translational states into signals that can be decoded by downstream disassembly and degradation machinery through site-specific ubiquitination of ribosomal proteins, thereby serving as a crucial coordinator of translation surveillance and proteostasis maintenance [5,6,7]. In addition to its canonical role in RQC, ZNF598 has been implicated in the translational repression of defective mRNAs [8,9], modulation of inflammatory signaling [10], antiviral defense [11,12,13], and control of aberrant translation products associated with neurodegenerative diseases [14,15], suggesting that its biological functions extend beyond classical ribosome quality control.

This review summarizes the structural basis of ZNF598, its molecular roles in the surveillance of aberrant translation, its regulatory network, and its potential involvement in human disease. Major unresolved questions and future directions are also discussed.

## 2. Structural Features and Canonical Mechanisms of ZNF598

### 2.1. Structural Organization and Molecular Characteristics of ZNF598

**Figure 1:**
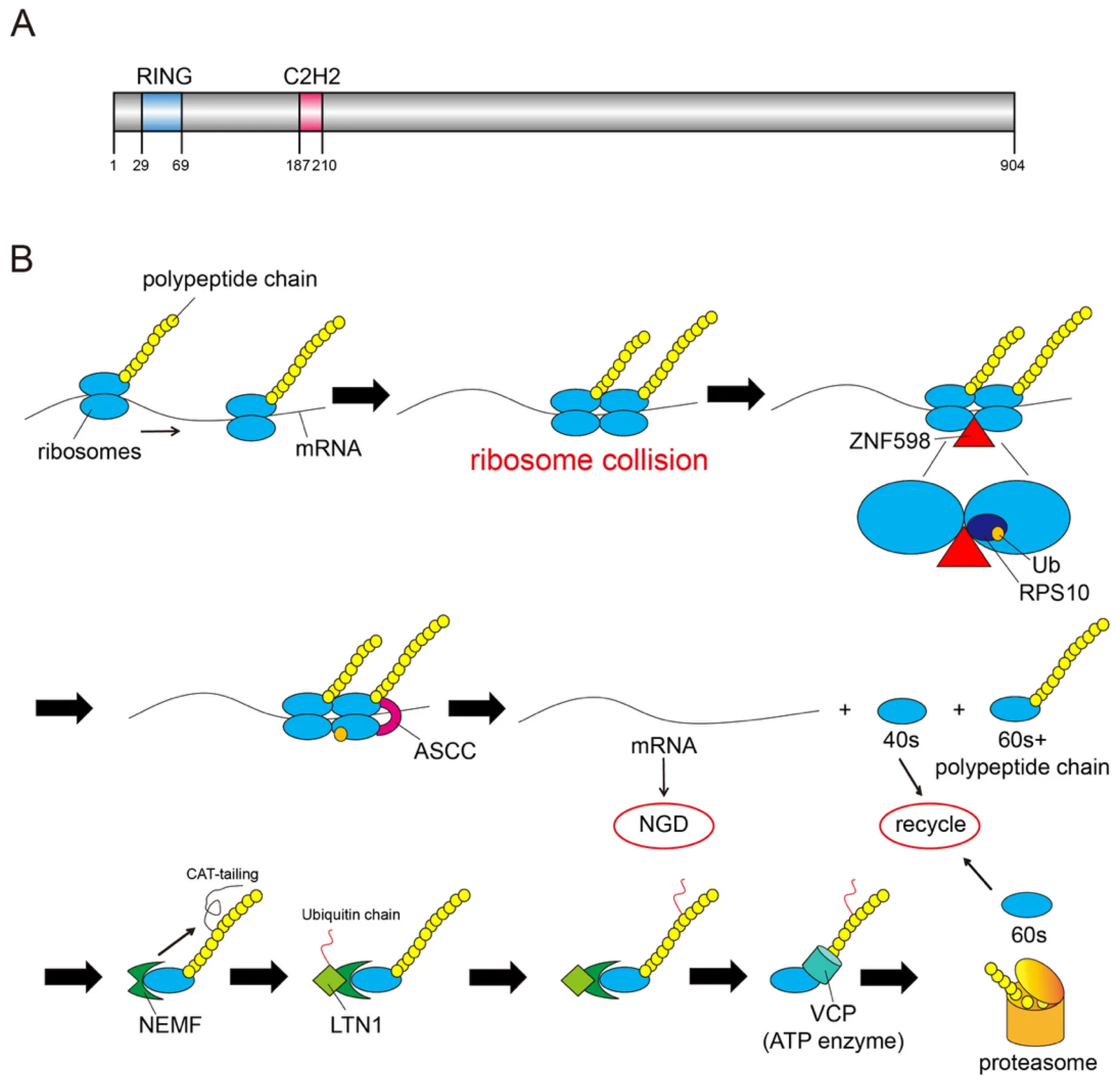
Domain architecture of ZNF598 and the canonical ribosome-associated quality control (RQC) pathway. (**A**) Schematic representation of the domain architecture of human ZNF598 (NM_178167.4). ZNF598 contains a RING finger domain and a C2H2 zinc-finger domain. (**B**) The canonical RQC pathway. Upon ribosome collision and translational stalling, ZNF598 recognizes collided ribosomes and ubiquitinates the 40S ribosomal proteins RPS10. The ubiquitinated 40S subunit subsequently recruits the ASC-1 complex (ASCC), which promotes the dissociation of the stalled 80S ribosome. Following ribosome splitting, the mRNA and 40S subunit are recycled, whereas the 60S large subunit remains associated with the peptidyl-tRNA-containing nascent polypeptide. The adaptor protein NEMF then binds to the stalled 60S subunit and serves as a platform for the recruitment of LTN1, forming the NEMF–LTN1 complex. In addition, NEMF mediates C-terminal alanine/threonine tailing (CAT-tailing) of the nascent chain, thereby exposing internal lysine residues and facilitating their accessibility for ubiquitination. As an E3 ubiquitin ligase, LTN1 catalyzes the conjugation of ubiquitin chains to the exposed lysine residues, marking the nascent polypeptide for degradation. The ubiquitinated polypeptide is subsequently extracted from the 60S subunit by the ATPase VCP/p97 and delivered to the proteasome for degradation. Finally, the cleared 60S ribosomal subunit is recycled for subsequent rounds of translation.

ZNF598 is a zinc finger-containing E3 ubiquitin ligase that is predominantly localized in the cytoplasm and closely associated with translation-related complexes in mammalian cells. Its primary structure contains a RING domain and a C2H2-type zinc finger motif, which are proposed to contribute to molecular interactions and substrate recognition, whereas the RING domain mediates ubiquitin transfer [5,6]. The RING domain constitutes the catalytic core of the E3 ligase, enabling the recruitment of E2 ubiquitin-conjugating enzymes and the transfer of ubiquitin to substrate proteins. Other structural regions are thought to contribute to ribosome binding, RNA-associated interactions, and the assembly of regulatory protein complexes (Figure 1A).

Emerging evidence suggests that ZNF598 is not merely a conventional E3 ubiquitin ligase but may also possess RNA-associated binding properties. Garzia et al. showed that ZNF598 can be crosslinked to mRNA, rRNA, and tRNA, suggesting that it may couple ribosome sensing with signal transduction during aberrant translation surveillance [4]. Nevertheless, the precise division of labor among individual structural domains in RNA binding, recognition of collided ribosomes, and substrate selection remains incompletely defined. Thus, the structure–function relationship of ZNF598 is still being actively refined.

### 2.2. Recognition of Collided Ribosomes and Ubiquitination of Ribosomal Proteins

When a leading ribosome stalls during elongation owing to mRNA defects or other perturbations, trailing ribosomes may collide with it and form collided ribosome complexes. Juszkiewicz et al. demonstrated that ZNF598 preferentially recognizes such collided conformations and interprets them as signals of aberrant translation [4]. This finding established the classical model in which ZNF598 functions as a sensor of collided ribosomes.

Upon the recognition of aberrant translation complexes, ZNF598 catalyzes site-specific ubiquitination of 40S ribosomal proteins, with RPS10/RPS20 being the most extensively characterized substrate, together with additional 40S proteins including uS3 and uS5 identified in mammalian and yeast systems. These modifications do not directly trigger ribosome degradation; rather, they appear to function as molecular marks that facilitate the recruitment of downstream factors involved in ribosome disassembly and quality control. Narita et al. further showed that K63-linked polyubiquitination of RPS10 in mammalian cells is closely associated with hRQT/ASCC-mediated ribosome splitting, indicating that ribosomal protein ubiquitination serves as a mechanistic bridge between aberrant translation recognition and downstream execution [4,6,7].

### 2.3. Coupling Ribosome Splitting to Downstream Quality Control

ZNF598-mediated ubiquitination of ribosomal proteins promotes the action of ribosome-splitting factors such as the ASC-1 complex (ASCC). The ASCC complex is composed of TRIP4, ASCC2, and ASCC3. TRIP4 acts as a scaffold component that facilitates complex assembly, while ASCC2 contains a CUE domain (coupling of ubiquitin conjugation to ER degradation) that specifically recognizes ubiquitin modifications and mediates the stable association of ASCC with ubiquitinated ribosomes. Following recruitment, the ATPase/helicase component ASCC3 promotes 80S ribosome splitting, resulting in the separation of the 40S subunit from the 60S subunit carrying the nascent polypeptide chain [16,17]. Following subunit dissociation, the 60S subunit carrying peptidyl-tRNA enters the canonical 60S–RQC pathway, whereas the released 40S subunit and associated mRNA are directed toward recycling or additional RNA surveillance pathways.

During the 60S–RQC phase, NEMF and LTN1 form the core machinery responsible for recognizing and processing aberrant nascent chains retained on the 60S subunit. NEMF promotes events such as CAT-tailing, whereas LTN1 mediates ubiquitination of the nascent chain, which is subsequently extracted from the ribosome by factors including VCP/p97 and delivered to the proteasome for degradation. Meanwhile, defective mRNAs may be further processed by NGD and related decay pathways, thereby reducing the likelihood of repeated aberrant translation from the same defective template [18,19,20].

Collectively, ZNF598 does not execute the entire quality control program by itself. Rather, it functions as a pivotal initiator of aberrant translation recognition and signal amplification, coordinating multiple downstream modules involved in ribosome splitting, nascent chain clearance, and defective mRNA turnover (Figure 1B).

## 3. ZNF598 in Specialized Translational Stalling and Translational Repression

### 3.1. Role in Poly(A)-Associated Translational Stalling

**Figure 2:**
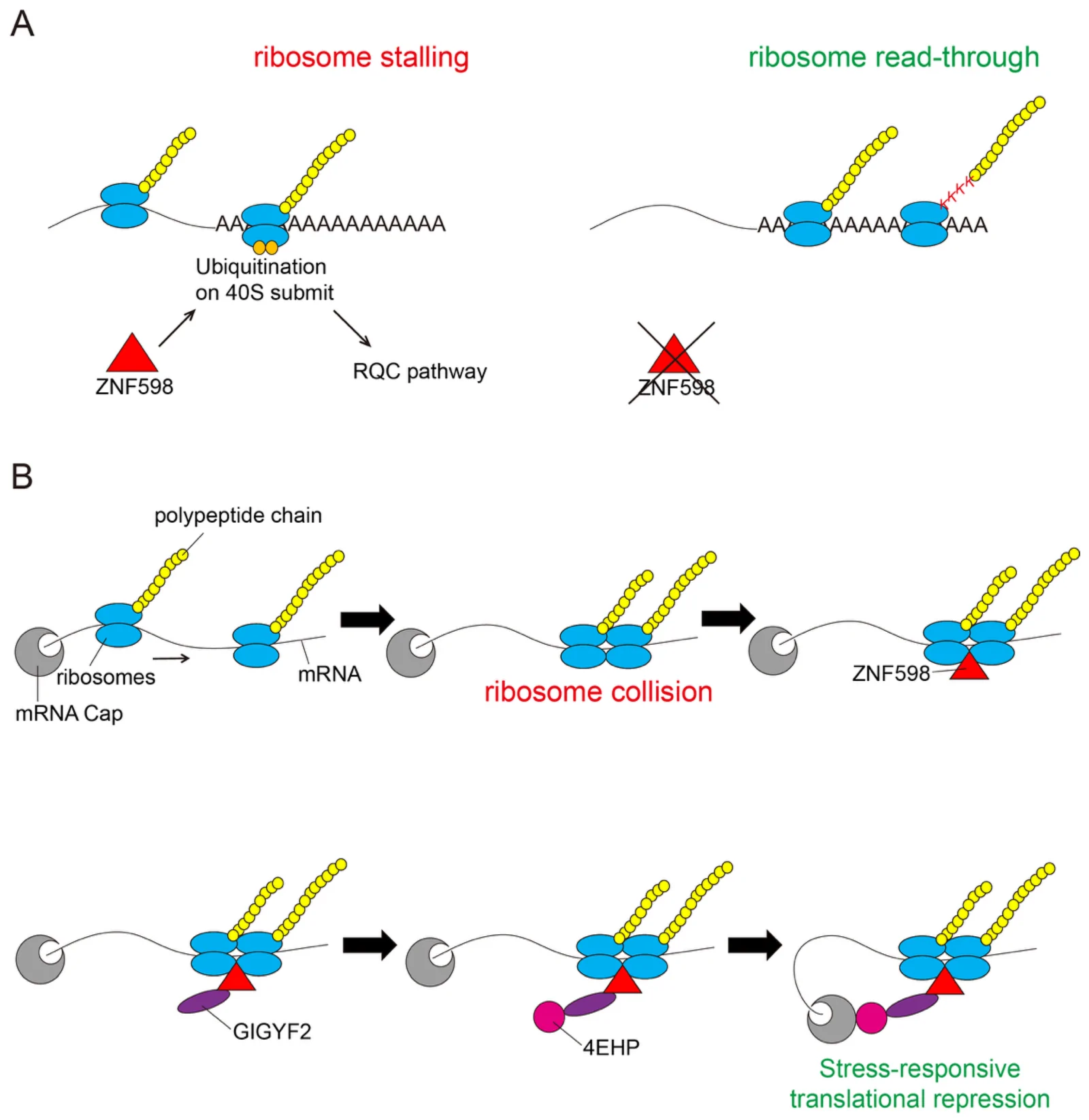
Dual roles of ZNF598 in translational repression and ribosome-associated quality control. (**A**) Translation of internal poly(A) sequences or the poly(A) tail of nonstop mRNAs activates ZNF598, which catalyzes the monoubiquitination of RPS10 at Lys138 and Lys139 on the 40S ribosomal subunit. This ubiquitination event promotes ribosome stalling and triggers the ribosome-associated quality control (RQC) pathway. In the absence of ZNF598, RPS10 is not monoubiquitinated, resulting in defective ribosome stalling and continued translation through poly(A) sequences (poly(A) readthrough). (**B**) Besides mediating the RQC pathway, ZNF598 functions as a scaffold protein that recruits GIGYF2 and 4EHP to defective mRNAs through direct protein–protein interactions. Cap binding by 4EHP inhibits translation initiation complex assembly, thereby preventing additional ribosomes from initiating translation on the aberrant mRNA.

Poly(A)-associated stalling represents one of the classical experimental models for investigating ZNF598 function. When ribosomes translate prematurely polyadenylated mRNAs or mRNAs lacking proper termination signals and extend into poly(A) sequences, translation becomes highly prone to abnormal stalling. Available studies have shown that site-specific ribosomal protein ubiquitination mediated by ZNF598 is required for stable terminal stalling during poly(A) translation and for subsequent activation of the RQC pathway [5,21]. In this setting, modification of RPS10 significantly influences whether ribosomes adopt a stable stalled state and how they are subsequently processed (Figure 2A).

Garzia et al. further demonstrated that ZNF598 is not only an E3 ubiquitin ligase, but also an RNA-associated protein with intrinsic RNA-binding properties. Its zinc finger domains enable interactions with multiple RNA species, including tRNAs, mRNAs, and rRNAs, with a particular preference for tRNA Lys (UUU). During the translation of poly(A) sequences, ribosomes encounter repeated lysine codons, leading to the accumulation of Lys-tRNA in the A site. ZNF598 can directly sense the accumulation of Lys-tRNA caused by poly(A)-induced translational stalling, subsequently promoting ubiquitination of the ribosomal protein RPS10 and initiating the ribosome-associated quality control (RQC) pathway [5]. These observations support the notion that ZNF598 may integrate both sensing and effector functions in poly(A)-associated translational quality control. However, the causal relationship between its RNA-binding behavior and ribosome recognition remains to be clarified.

### 3.2. Role in Aberrant Repeat-Associated Translation

ZNF598 has also been implicated in the handling of translation products generated from expanded repeat sequences. In C9ORF72-associated amyotrophic lateral sclerosis/frontotemporal dementia (ALS/FTD), expanded repeat sequences give rise to neurotoxic dipeptide repeat proteins such as poly(GR). Previous studies have shown that ZNF598 can limit the production of specific toxic repeat-derived proteins during translation and promote their clearance through the RQC pathway, thereby alleviating neurotoxicity to some extent. However, the magnitude and direction of ZNF598-mediated regulation may vary depending on the repeat sequence, reading frame, and experimental model. Accordingly, its protective role should be interpreted with appropriate caution and within a defined experimental context [14,15].

### 3.3. Role in Translational Repression of Defective mRNAs

In addition to its canonical role in RQC, ZNF598 contributes to the translational repression of defective mRNAs. Current evidence suggests that when certain abnormal mRNAs induce ribosome stalling, ZNF598 functions as a scaffold/adaptor that facilitates the recruitment of GIGYF2 and 4EHP to the affected transcripts, thereby suppressing cap-dependent translation initiation and preventing additional ribosomes from loading onto the same defective template. By recruiting the GIGYF2–4EHP complex, ZNF598 inhibits eIF4F complex assembly and attenuates translation initiation [8,9]. This emerging mechanism broadens the functional landscape of ZNF598, positioning it not merely as a ribosome-associated quality control factor but as a key regulator of translational homeostasis. This mechanism operates in parallel with ribosome splitting and nascent chain degradation, together forming a multilayered defense against defective mRNAs. This dual function raises an important mechanistic question: why does a ribosome-associated quality control factor also regulate translation initiation? One possible explanation is that ZNF598 serves a complementary role in maintaining translational balance. Extensive ribosome collisions can disrupt proteome homeostasis and activate RQC pathways to eliminate stalled translation complexes, whereas aberrant mRNAs may represent an upstream cause of translational imbalance. By simultaneously triggering GIGYF2/4EHP-mediated translational repression, ZNF598 may prevent the excessive recruitment of additional ribosomes onto problematic transcripts. Thus, ZNF598 may function as a molecular switch that coordinates the balance between translation maintenance and translation repression (Figure 2B).

In this context, ZNF598 appears to function less as a simple ubiquitin ligase and more as a molecular bridge linking aberrant translation recognition to the translational repression machinery. This functional expansion broadens the current view of ZNF598 biology and further underscores that translational quality control is not a linear pathway but an integrated network of parallel and interconnected regulatory branches.

### 3.4. Post-Transcriptional Regulation of Inflammation-Related mRNAs

Emerging evidence further suggests that ZNF598 may form functional complexes with GIGYF1/2 and 4EHP and participate in regulating the translation and stability of inflammation-related mRNAs. In some studies, ZNF598 has been described as having functions reminiscent of RNA-binding regulatory proteins, suppressing the excessive expression of inflammatory mediators under specific cellular conditions. However, compared with its well-established role in collided ribosome surveillance, the target spectrum, mechanistic hierarchy, and context specificity of ZNF598 in post-transcriptional inflammatory regulation remain insufficiently understood and warrant further investigation [10,22].

Tristetraprolin (TTP) is an RNA-binding protein that serves as an adaptor to recruit the GIGYF2–4EHP translational repression complex to inflammation-associated mRNAs, thereby suppressing the expression of pro-inflammatory factors. Given the structural similarity between ZNF598 and TTP, it is possible that ZNF598 may also interact with the GIGYF2–4EHP complex and contribute to the translational repression of specific mRNAs in a similar manner [10]. ZNF598 also has the function of regulating the expression of inflammatory factors.

## 4. Regulatory Network of ZNF598

### 4.1. Upstream Activating Signals

Current evidence suggests that any perturbation that increases the probability of translational stalling or the formation of aberrant ribosomal conformations may promote ZNF598-dependent responses. Such perturbations include positively charged amino acid stretches, enrichment of rare codons, mRNA secondary structures, termination defects, poly(A) translation, cellular stress, and certain viral infections [4,6,21,23]. Among these, ribosome collision remains the best-characterized and most directly supported activating context. However, this does not imply that all ZNF598-dependent events are initiated through an identical upstream mechanism [16] (Table 1).

### 4.2. Interacting Factors

The function of ZNF598 depends on coordinated interactions with multiple regulatory factors. In canonical RQC, ASCC, NEMF, LTN1, and VCP/p97 constitute major downstream effectors [16,17,18]. In the translational repression branch, GIGYF2 and 4EHP are among its key functional partners [8,9,10,22]. In addition, deubiquitinating enzymes may influence the efficiency of quality control by modulating ribosomal protein ubiquitination or ZNF598 stability. For example, USP9X has been implicated in the regulation of ribosomal stalling, whereas USP10 participates in deubiquitination and the rescue of damaged 40S ribosomal subunits [30,31]. These findings indicate that the ZNF598 regulatory network is not a unidirectional cascade but rather a finely tuned system balanced by both ubiquitination and deubiquitination (Table 1).

Under mitochondrial stress and other specific conditions, the CNOT4–ZNF598 axis has also been implicated in translational stress responses and tissue homeostasis [32]. This suggests that ZNF598 may assemble with distinct cofactors under different stress conditions, thereby producing context-dependent functional outcomes.

### 4.3. Substrate Specificity and Signal Integration

ZNF598 does not respond uniformly to all forms of translational slowdown. Available studies indicate that its substrate selectivity is closely related to ribosomal conformation, sequence features of the mRNA, the duration of translational pausing, and the assembly state of accessory factors [4,7,16]. Accordingly, ZNF598 may be more appropriately viewed as an integrative node for aberrant translational states. Rather than responding mechanically to a single signal, it appears to integrate multiple forms of stalling-associated information and determine whether and how downstream quality control pathways should be engaged.

## 5. Roles of ZNF598 in Physiology and Disease

### 5.1. Maintenance of Proteostasis and Neuronal Integrity

Neuronal development requires a highly maintained intracellular homeostasis, among which the precise regulation of protein synthesis is particularly important. Therefore, the disruption of translational homeostasis may have profound effects on neuronal development and function. Current studies have demonstrated that the impairment of ZNF598-mediated quality control increases the production of aberrant translation products, particularly repeat-associated toxic polypeptides, thereby exacerbating neuronal stress and neurodegenerative changes [14]. Given its central role in maintaining translational quality control, ZNF598 has emerged as a potential regulator of neuronal proteostasis. Loss of ZNF598 activity may lead to ribosome stalling, the abnormal accumulation of nascent peptide products, and increased proteotoxic stress, ultimately compromising neuronal survival and synaptic circuit formation. This possibility is particularly relevant to neurodevelopmental disorders and neurodegenerative diseases, in which translational dysregulation and impaired proteostasis are frequently observed. Alterations in ribosome-associated quality control may affect neuronal differentiation, synaptic protein synthesis, and axonal maintenance [33,34] (Table 1).

However, the mechanisms by which ZNF598 dysfunction directly contributes to human neurological disorders remain incompletely understood. Unlike some well-established neurodevelopmental disease-associated genes, pathogenic ZNF598 variants are currently rare and require further systematic investigation. Future studies integrating human genetic datasets, patient-derived models, and neuronal functional analyses will help elucidate the contribution of ZNF598-mediated translational surveillance to disease pathogenesis.

### 5.2. Viral Infection and Innate Immune Regulation

The role of ZNF598 in viral infection appears to be multifaceted. Recent studies have revealed that viruses can actively remodel the ZNF598-dependent ribosome-associated quality control (RQC) pathway to optimize their own translation processes. Viral infections have provided insights into the context-dependent functions of ZNF598-mediated RQC. Epstein–Barr virus (EBV) encodes a viral deubiquitinase, BPLF1, which counteracts ZNF598-mediated ubiquitination of 40S ribosomal proteins, thereby suppressing downstream RQC activity and preventing the degradation of stalled translation products. Impaired RQC function further activates a GCN2-dependent integrated stress response (ISR), which reprograms cellular translation and promotes the selective translation of viral transcripts. In contrast, vaccinia virus infection exploits RQC activity to maintain efficient viral protein synthesis under conditions of high translational stress, thereby enhancing its own translation efficiency. These findings highlight the critical role of ZNF598-dependent RQC in balancing host proteostasis defense and viral translational adaptation [23,24,25] (Table 1).

In addition, ZNF598 has been reported to be associated with interferon-stimulated gene expression, cGAS-related responses, and RIG-I signaling [11,12,13,35]. These observations suggest that the biological significance of ZNF598 extends beyond protein quality control and may place it at the interface between translational stress and innate immunity. Nevertheless, most of these findings derive from specific experimental systems, and their broader generalizability and mechanistic unity remain to be established.

### 5.3. Tumor-Related Implications

Cancer cells, due to their rapid proliferation and metabolic reprogramming, exhibit a significant increase in protein synthesis demand. This enhanced translational activity inevitably leads to proteotoxic stress, including ribosome collisions and defects in nascent polypeptides. In cancer biology, the RQC pathway has been proposed to influence the capacity of tumor cells to adapt to hypoxia, endoplasmic reticulum stress, metabolic stress, and chemotherapy-induced translational perturbation [26,27,28]. Although there is currently no direct evidence linking ZNF598 mutations to human cancers, emerging studies suggest that ZNF598-dependent ribosome-associated quality control (RQC) may influence the responses of cancer cells to translational stress. For example, Chatterjee et al. demonstrated that the chemotherapeutic agent 5-fluorouracil (5-FU) induces pharmacological ribosome collisions, thereby activating the RQC pathway. Impairment of RQC function increases drug-induced cytotoxicity and enhances sensitivity to chemotherapy [29]. From this perspective, ZNF598, as a key initiator of RQC, may theoretically contribute to tumor cell tolerance to translational stress. However, current evidence regarding the expression pattern, causal role, and clinical relevance of ZNF598 across different tumor types remains limited. It is therefore premature to classify ZNF598 simply as either a stable oncogenic factor or a tumor suppressor (Table 1).

### 5.4. Development and Tissue Homeostasis

Studies in animal models suggest that the loss of ZNF598 may affect organismal development and tissue homeostasis. For example, in zebrafish, mutation of ZNF598 has been associated with phenotypes such as reduced body size and decreased survival, indicating an important developmental function [28]. Together with findings implicating the CNOT4–ZNF598 pathway under mitochondrial stress, these observations suggest that ZNF598 may be particularly important for maintaining homeostasis in tissues with high translational demand, although the underlying mechanisms remain to be elucidated [32].

## 6. Conclusions and Perspectives

As a central component of the eukaryotic translational quality control network, ZNF598 plays a critical role in the recognition of aberrant translation complexes, ubiquitination of ribosomal proteins, and coordination of downstream quality control pathways. Studies of its canonical function have established a coherent framework in which ZNF598 senses aberrant translational states, particularly ribosome collision-associated conformations, and converts them into signals that can be decoded by downstream machinery through site-specific ubiquitination [4,6,17]. At the same time, more recent work indicates that the functional scope of ZNF598 may extend well beyond classical RQC to include translational repression, inflammatory regulation, viral infection, and stress adaptation.

Several important questions remain unresolved. First, the principles by which ZNF598 distinguishes among different aberrant translational states have not yet been fully unified, and the relative contributions of collided ribosomes versus non-collided abnormal complexes remain to be clarified [16]. Second, the specific roles of individual ZNF598 domains in RNA binding, substrate selection, and cofactor recruitment are still not supported by sufficient high-resolution structural and functional evidence [5]. Third, the regulatory modes of ZNF598 may vary across tissues, developmental stages, and stress conditions, and its physiological functional landscape has yet to be systematically defined.

From a translational perspective, ZNF598-associated pathways may hold therapeutic promise. For example, enhancement of aberrant translation product clearance may prove beneficial in neurodegenerative disease, whereas targeting RQC nodes may alter survival programs in tumor models that rely on adaptation to translational stress. For example, modulation of the GIGYF2/4EHP-mediated translational repression pathway or specific RQC components may allow for more precise control of pathological translation stress while preserving basal quality-control functions. However, because ZNF598 functions within an evolutionarily conserved process tightly linked to basal translational homeostasis, any therapeutic strategy targeting this pathway must carefully consider tissue specificity, safety, and potential off-target effects.

In summary, ZNF598 occupies a central position at the intersection of translation surveillance, proteostasis, and stress response. Continued advances in structural biology, in vivo imaging, ribosome profiling, and disease modeling are expected to provide deeper insight into its precise mechanisms of action and strengthen the theoretical basis for understanding translational quality control and developing interventions for related diseases.

## 7. Literature Search Strategy

A comprehensive literature search was conducted using the PubMed, Web of Science, and Google Scholar databases to identify relevant studies regarding the biological functions and mechanisms of ZNF598. The search was performed using combinations of keywords including “ZNF598”, “zinc finger protein 598”, “ribosome-associated quality control”, “RQC”, “ribosome collision”, “ribosome stalling”, “ribosomal ubiquitination”, “ASCC complex”, “GIGYF2”, “4EHP”, “translational repression”, “RNA binding”, “immune regulation”, “viral infection”, “cancer”, and “neurodegeneration”. Literature published up to June 2026 was considered, with particular emphasis on mechanistic studies, molecular interaction studies, and disease-related investigations.

Studies were selected based on their relevance to the functional mechanisms of ZNF598, including ribosome collision sensing, ribosomal protein ubiquitination, recruitment of downstream RQC factors, translational regulation, RNA-associated functions, and emerging roles in physiological and pathological contexts. Primary research articles were prioritized, while relevant review articles were used to provide historical background and broader conceptual frameworks. Studies lacking direct relevance to ZNF598 biology or without sufficient mechanistic information were excluded.

This narrative review integrates findings from genetic, biochemical, structural, and cellular studies to provide a comprehensive overview of the current understanding of ZNF598-mediated translational quality control and its implications in human health and disease.

## Figures and Tables

**Table 1:** Functional landscape of ZNF598-mediated pathways.

Function	ZNF598 Partners	Molecular Event	Representative Studies
Ribosome collision sensing	RPS10/RPS20	Ubiquitination	Juszkiewicz et al. [4,5,6,7]
Ribosome rescue	ASCC complex	Ubiquitin-dependent recruitment	Wang et al. [13,16,17]
Nascent peptide degradation	LTN1/NEMF/VCP	Degradation	Brandman et al. [1,18,19,20]
Translation repression	GIGYF2/4EHP	Inhibition of initiation	Weber et al. [8,9]
RNA-associated regulation	RNA-associated factors	RNA recognition	Garzia et al. [5,21]
Viral infection	BPLF1, viral factors	RQC remodeling	Liu et al. [23,24,25]
Pharmacological stress	RQC machinery	Collision resolution	Chatterjee et al. [26,27,28,29]
Neurobiology	local translation machinery	Translation surveillance	emerging
Cancer	RQC components	Stress adaptation	emerging

## Data Availability

No new data were created or analyzed in this study. Data sharing is not applicable to this article.

## Abbreviations

ZNF598	Zinc Finger Protein 598
RQC	Ribosome-Associated Quality Control
GIGYF2	GRB10 Interacting GYF Protein 2
NGD	No-Go Decay
NSD	Non-Stop Decay
RPS10	Ribosomal Protein S10
ASCC	ASC-1 Complex
CUE	Coupling of Ubiquitin Conjugation to ER Degradation
LTN1	LISTERIN E3 Ubiquitin Protein Ligase 1
NEMF	Nuclear Export Mediator Factor
VCP	Valosin Containing Protein
ALS/FTD	Amyotrophic Lateral Sclerosis/Frontotemporal Dementia
4EHP	Eukaryotic Translation Initiation Factor 4E Homologous Protein
TTP	Tristetraprolin
USP9X	Ubiquitin Specific Peptidase 9 X-Linked
USP10	Ubiquitin Specific Peptidase 10
CNOT4	CCR4-NOT Transcription Complex Subunit 4
EBV	Epstein–Barr Virus
BPLF1	Bamhi P Leftward Frame 1 Deubiquitinating Enzyme
5-FU	5-Fluorouracil

## References

[1] Brandman O., Hegde R.S. (2016). Ribosome-associated protein quality control. Nat. Struct. Mol. Biol..

[2] Brandman O., Stewart-Ornstein J., Wong D., Larson A., Williams C.C., Li G.W., Zhou S., King D., Shen P.S., Weibezahn J. (2012). A ribosome-bound quality control complex triggers degradation of nascent peptides and signals translation stress. Cell.

[3] De La Cruz A.C., Tisdale G., Nakayama E., Huang Z., Sinha N.K., Green R., Wu B. (2025). Single-protein/RNA imaging reveals ZNF598 as a limiting factor in resolving collided ribosomes. EMBO J..

[4] Juszkiewicz S., Chandrasekaran V., Lin Z., Kraatz S., Ramakrishnan V., Hegde R.S. (2018). ZNF598 Is a Quality Control Sensor of Collided Ribosomes. Mol. Cell.

[5] Garzia A., Jafarnejad S.M., Meyer C., Chapat C., Gogakos T., Morozov P., Amiri M., Shapiro M., Molina H., Tuschl T. (2017). The E3 ubiquitin ligase and RNA-binding protein ZNF598 orchestrates ribosome quality control of premature polyadenylated mRNAs. Nat. Commun..

[6] Sundaramoorthy E., Leonard M., Mak R., Liao J., Fulzele A., Bennett E.J. (2017). ZNF598 and RACK1 Regulate Mammalian Ribosome-Associated Quality Control Function by Mediating Regulatory 40S Ribosomal Ubiquitylation. Mol. Cell.

[7] Narita M., Denk T., Matsuo Y., Sugiyama T., Kikuguchi C., Ito S., Sato N., Suzuki T., Hashimoto S., Machová I. (2022). A distinct mammalian disome collision interface harbors K63-linked polyubiquitination of uS10 to trigger hRQT-mediated subunit dissociation. Nat. Commun..

[8] Weber R., Chung M.Y., Keskeny C., Zinnall U., Landthaler M., Valkov E., Izaurralde E., Igreja C. (2020). 4EHP and GIGYF1/2 Mediate Translation-Coupled Messenger RNA Decay. Cell Rep..

[9] Hickey K.L., Dickson K., Cogan J.Z., Replogle J.M., Schoof M., D’Orazio K.N., Sinha N.K., Hussmann J.A., Jost M., Frost A. (2020). GIGYF2 and 4EHP Inhibit Translation Initiation of Defective Messenger RNAs to Assist Ribosome-Associated Quality Control. Mol. Cell.

[10] Tollenaere M.A.X., Tiedje C., Rasmussen S., Nielsen J.C., Vind A.C., Blasius M., Batth T.S., Mailand N., Olsen J.V., Gaestel M. (2019). GIGYF1/2-Driven Cooperation between ZNF598 and TTP in Posttranscriptional Regulation of Inflammatory Signaling. Cell Rep..

[11] DiGiuseppe S., Rollins M.G., Bartom E.T., Walsh D. (2018). ZNF598 Plays Distinct Roles in Interferon-Stimulated Gene Expression and Poxvirus Protein Synthesis. Cell Rep..

[12] Wan L., Juszkiewicz S., Blears D., Bajpe P.K., Han Z., Faull P., Mitter R., Stewart A., Snijders A.P., Hegde R.S. (2021). Translation stress and collided ribosomes are co-activators of cGAS. Mol. Cell.

[13] Wang G., Kouwaki T., Okamoto M., Oshiumi H. (2019). Attenuation of the Innate Immune Response against Viral Infection Due to ZNF598-Promoted Binding of FAT10 to RIG-I. Cell Rep..

[14] Park J., Lee J., Kim J.H., Lee J., Park H., Lim C. (2021). ZNF598 co-translationally titrates poly(GR) protein implicated in the pathogenesis of C9ORF72-associated ALS/FTD. Nucleic Acids Res..

[15] Latallo M.J., Wang S., Dong D., Nelson B., Livingston N.M., Wu R., Zhao N., Stasevich T.J., Bassik M.C., Sun S. (2023). Single-molecule imaging reveals distinct elongation and frameshifting dynamics between frames of expanded RNA repeats in C9ORF72-ALS/FTD. Nat. Commun..

[16] Miścicka A., Bulakhov A.G., Kuroha K., Zinoviev A., Hellen C.U.T., Pestova T.V. (2024). Ribosomal collision is not a prerequisite for ZNF598-mediated ribosome ubiquitination and disassembly of ribosomal complexes by ASCC. Nucleic Acids Res..

[17] Juszkiewicz S., Speldewinde S.H., Wan L., Svejstrup J.Q., Hegde R.S. (2020). The ASC-1 Complex Disassembles Collided Ribosomes. Mol. Cell.

[18] Joazeiro C.A.P. (2017). Ribosomal Stalling During Translation: Providing Substrates for Ribosome-Associated Protein Quality Control. Annu. Rev. Cell Dev. Biol..

[19] Shoemaker C.J., Eyler D.E., Green R. (2010). Dom34:Hbs1 promotes subunit dissociation and peptidyl-tRNA drop-off to initiate no-go decay. Science.

[20] Shoemaker C.J., Green R. (2012). Translation drives mRNA quality control. Nat. Struct. Mol. Biol..

[21] Juszkiewicz S., Hegde R.S. (2017). Initiation of Quality Control during Poly(A) Translation Requires Site-Specific Ribosome Ubiquitination. Mol. Cell.

[22] Morita M., Ler L.W., Fabian M.R., Siddiqui N., Mullin M., Henderson V.C., Alain T., Fonseca B.D., Karashchuk G., Bennett C.F. (2012). A novel 4EHP-GIGYF2 translational repressor complex is essential for mammalian development. Mol. Cell. Biol..

[23] Liu J., Nagy N., Ayala-Torres C., Aguilar-Alonso F., Morais-Esteves F., Xu S., Masucci M.G. (2023). Remodeling of the ribosomal quality control and integrated stress response by viral ubiquitin deconjugases. Nat. Commun..

[24] Liu J., Nagy N., Ayala-Torres C., Bleuse S., Aguilar-Alonso F., Larsson O., Masucci M.G. (2025). The Epstein-Barr virus deubiquitinase BPLF1 regulates stress-induced ribosome UFMylation and reticulophagy. Autophagy.

[25] Sundaramoorthy E., Ryan A.P., Fulzele A., Leonard M., Daugherty M.D., Bennett E.J. (2021). Ribosome quality control activity potentiates vaccinia virus protein synthesis during infection. J. Cell Sci..

[26] Pelletier J., Thomas G., Volarević S. (2018). Ribosome biogenesis in cancer: New players and therapeutic avenues. Nat. Rev. Cancer.

[27] Hanahan D. (2022). Hallmarks of Cancer: New Dimensions. Cancer Discov..

[28] Ishibashi K., Shichino Y., Han P., Wakabayashi K., Mito M., Inada T., Kimura S., Iwasaki S., Mishima Y. (2024). Translation of zinc finger domains induces ribosome collision and Znf598-dependent mRNA decay in zebrafish. PLoS Biol..

[29] Chatterjee S., Naeli P., Onar O., Simms N., Garzia A., Hackett A., Coyle K., Harris Snell P., McGirr T., Sawant T.N. (2024). Ribosome Quality Control mitigates the cytotoxicity of ribosome collisions induced by 5-Fluorouracil. Nucleic Acids Res..

[30] Clancy A., Heride C., Pinto-Fernández A., Elcocks H., Kallinos A., Kayser-Bricker K.J., Wang W., Smith V., Davis S., Fessler S. (2021). The deubiquitylase USP9X controls ribosomal stalling. J. Cell Biol..

[31] Meyer C., Garzia A., Morozov P., Molina H., Tuschl T. (2020). The G3BP1-Family-USP10 Deubiquitinase Complex Rescues Ubiquitinated 40S Subunits of Ribosomes Stalled in Translation from Lysosomal Degradation. Mol. Cell.

[32] Geng J., Li S., Li Y., Wu Z., Bhurtel S., Rimal S., Khan D., Ohja R., Brandman O., Lu B. (2024). Stalled translation by mitochondrial stress upregulates a CNOT4-ZNF598 ribosomal quality control pathway important for tissue homeostasis. Nat. Commun..

[33] Darnell J.C., Klann E. (2013). The translation of translational control by FMRP: Therapeutic targets for FXS. Nat. Neurosci..

[34] Wang S., Sun S. (2023). Translation dysregulation in neurodegenerative diseases: A focus on ALS. Mol. Neurodegener..

[35] Mah M.M., Roverato N., Groettrup M. (2020). Regulation of Interferon Induction by the Ubiquitin-Like Modifier FAT10. Biomolecules.

